# Null mutation of the endothelin receptor type B gene causes embryonic death in the GK rat

**DOI:** 10.1371/journal.pone.0217132

**Published:** 2019-06-06

**Authors:** Jinxi Wang, Ruihua Dang, Yoshiki Miyasaka, Kousuke Hattori, Daisuke Torigoe, Tadashi Okamura, Hassan T. Tag-EI-Din-Hassan, Masami Morimatsu, Tomoji Mashimo, Takashi Agui

**Affiliations:** 1 Laboratory of Laboratory Animal Science and Medicine, Department of Applied Veterinary Sciences, Graduate School of Veterinary Medicine, Hokkaido University, Sapporo, Japan; 2 Institute of Experimental Animal Sciences, Graduate School of Medicine, Osaka University, Osaka, Japan; 3 College of Animal Science and Technology, Northwest A&F University, Yangling, Shaanxi, China; 4 Section of Animal Models, Department of Infectious Diseases, Research Institute, National Center for Global Health and Medicine, Tokyo, Japan; 5 Poultry Production Department, Faculty of Agriculture, Mansoura University, Mansoura, Egypt; National Institute of Genetics, JAPAN

## Abstract

The Hirschsprung disease (HSCR) is an inherited disease that is controlled by multiple genes and has a complicated genetic mechanism. HSCR patients suffer from various extents of constipation due to dysplasia of the enteric nervous system (ENS), which can be so severe as to cause complete intestinal obstruction. Many genes have been identified as playing causative roles in ENS dysplasia and HSCR, among them the endothelin receptor type B gene (*Ednrb*) has been identified to play an important role. Mutation of *Ednrb* causes a series of symptoms that include deafness, pigmentary abnormalities, and aganglionosis. In our previous studies of three rat models carrying the same spotting lethal (*sl*) mutation on *Ednrb*, the haplotype of a region on chromosome (Chr) 2 was found to be responsible for the differing severities of the HSCR-like symptoms. To confirm that the haplotype of the responsible region on Chr 2 modifies the severity of aganglionosis caused by *Ednrb* mutation and to recreate a rat model with severe symptoms, we selected the GK inbred strain, whose haplotype in the responsible region on Chr 2 resembles that of the rat strain in which severe symptoms accompany the *Ednrb*^*sl*^ mutation. An *Ednrb* mutation was introduced into the GK rat by crossing with F344-*Ednrb*^*sl*^ and by genome editing. The null mutation of *Ednrb* was found to cause embryonic death in F_2_ progeny possessing the GK haplotype in the responsible region on Chr 2. The results of this study are unexpected, and they provide new clues and animal models that promise to contribute to studies on the genetic regulatory network in the development of ENS and on embryogenesis.

## Introduction

The enteric nervous system (ENS), an important and complex part of the visceral nervous system in vertebrates, originates from neural crest cells and extends into the wall of the alimentary canal along the entire length of that canal [1]. Under the regulation of the ENS, the intestine is capable of motility, including contraction and relaxation, which causes the intestinal tract contents to be passed along and feces to be expelled from the anus. Hirschsprung disease (HSCR) is a common congenital disorder of the gastrointestinal tract that occurs in about 1/5000 live births [2]. It is characterized by a defective ENS caused by the incomplete migration, differentiation, and proliferation of neural crest cells in the gastrointestinal track in the early embryonic stage [3]. The affected portion of the intestines is always a continuous portion of the caudal gastrointestinal tract and obstructs the smooth passage of stool [4]. Patients with this disease suffer from constipation of varying severity, including complete obstruction, depending on the length of the aganglionic section [2,5,6].

In HSCR, the penetrance and phenotypes of aganglionosis segment length vary by gender and familial incidence. The variation in phenotypes is attributed to the complex genetic interactions between discovered and unrevealed susceptibility loci or modifier loci in different genetic backgrounds, which regulate the development of ENS [3,5]. A series of genetic studies have already implicated several genes, including the *RET* proto-oncogene [7], the endothelin receptor type B gene (*EDNRB*) [8–10], the endothelin-3 gene (*EDN3*) [11], the glial-cell-line-derived neurotrophic factor gene (*GDNF*) [12,13], the SRY-related HMG-box 10 gene (*SOX10*) [14], the neurturin gene (*NRTN*) [15], the endothelin converting enzyme 1 gene (*ECE1*) [16], the zinc finger homeobox 1B gene (*ZFHX1B*) [17], the paired-like homeobox 2B gene (*PHOX2B*) [18], the KIF1 binding protein gene (*KIF1BP*) [19], and the transcription factor 4 gene (*TCF4*) [16]. Of these, the *EDNRB* gene, which is involved in the EDN3/EDNRB signaling, is known to play a key role in the development of HSCR, as either the heterozygous or homozygous mutation of this gene is found in HSCR patients [10,20,21].

The spotting lethal (*sl*) mutation occurs naturally as a null mutation in the rat *Ednrb*, which displays a 301-bp deletion that leads to dysfunction of the corresponding protein [22]. In previous studies, we produced three inbred and congenic rat strains carrying the *sl* mutation on *Ednrb*: the AGH (aganglionosis Hokkaido)-*Ednrb*^*sl*^, the LEH (Long-Evans Hokkaido)-*Ednrb*^*sl*^, and the F344-*Ednrb*^*sl*^ [23]. The symptoms caused by this mutation differ according to the genetic background. The AGH-*Ednrb*^*sl*^ displayed very severe symptom of megacolon with a very low survival rate until weaning. The ratio of aganglionic length to large intestine length of this strain was more than 2.0. On the other hand, the LEH-*Ednrb*^*sl*^ and F344-*Ednrb*^*sl*^ displayed much milder symptom, and most of the F344-*Ednrb*^*sl*^ can survive with a small aganglionic region. The ratio of aganglionic length to large intestine length of the F344-*Ednrb*^*sl*^ was less than 0.5 [23]. Based on the differences in aganglionosis severity between the AGH-*Ednrb*^*sl*^ and the LEH-*Ednrb*^*sl*^ or F344-*Ednrb*^*sl*^, we performed quantitative trait locus (QTL) analysis on the two groups using the ratio of aganglonic intestine length to large intestine length as the quantitative trait, and we detected a genetic locus on chromosome (Chr) 2 as being responsible for this variation [24,25]. Comparisons of the sequence of genes located in the identified region of these three rat strains revealed different haplotypes between the rats that presented severe symptoms and those that presented mild symptoms [25], suggesting that different haplotypes of this region caused the variation in the symptoms of HSCR resulting from the *Ednrb*^*sl*^ mutation. However, the AGH-*Ednrb*^*sl*^, which displayed the most severe symptoms, went extinct for unknown reason. To continue the study of phenotypes of the *Ednrb*^*sl*^ mutation by creating a new rat model with the *Ednrb*^*sl*^ mutation that shows severe symptoms, the GK/Slc inbred rat strain was selected from database, as the haplotype of the responsible region is similar to that of the AGH rat. In this study, by preparing F_2_(F344 × GK)-*Ednrb*^*sl/sl*^ progeny and genome-edited GK rats, we found that the null mutation of the *Ednrb* gene causes the more serious symptoms of early embryonic death in GK rats, suggesting that the haplotype of Chr 2 plays an important role in regulating the phenotype of HSCR caused by the *Ednrb* mutation.

## Materials and methods

### Animals

F344, GK, and SD rats were purchased from Japan SLC, Inc. (Hamamatsu, Shizuoka, Japan). The F344-*Ednrb*^*sl/+*^ rats were obtained as described previously [23]. Male F344-*Ednrb*^*sl/+*^ rats were mated with female GK rats to produce the F_1_ generation, and the F_2_ progeny were generated by mating the heterozygous F_1_ offspring. The animals were maintained in specific pathogen-free conditions with feeding and drinking allowed *ad libitum*. All rats including 10-day-old F_2_ progeny and pregnant females were euthanized by inhalation of CO_2_ following the AVMA Guidelines for the Euthanasia of Animals: 2013 Edition. Fetuses collected from euthanized pregnant females were euthanized by decapitation. All research and experimental protocols were conducted according to the Regulation for the Care and Use of Laboratory Animals of Hokkaido University and Osaka University, and were approved by the Animal Care and Use Committee of Hokkaido University and Osaka University.

### Genotyping *Ednrb* and microsatellites

The *Ednrb* genotype was identified by primer set 1 (F-CCTCCTGGACTAGAGGTTCC and R-ACGACTTAGAAAGCTACACT). DNA samples were obtained from the tail tips or tissue by using a KAPA Express Extract DNA Extraction Kit (Kapa Biosystems, London, UK). PCR products were electrophoresed in 1.5% agarose gels to distinguish the wild alleles (517 bp) from the mutant alleles (216 bp). The microsatellite markers *D2Rat174*, *D2Rat201*, *D2Rat19*, and *D2Mgh21* were selected for identification of the haplotype of each F_2_ progeny in the responsible region, according to their location relative to the *Gdnf*, *Ptger4*, and *Slc45a2* on Chr 2 in the Rat Genome Database (RGD, https://rgd.mcw.edu). The positions of the genes and microsatellite markers were based on information from the RGD (assembly Rnor_6.0) (https://rgd.mcw.edu/wg/). All PCR products were electrophoresed in 10% polyacrylamide gels, stained with ethidium bromide, and photographed under an ultraviolet lamp.

### Knockout of *Ednrb* and *Rosa26* by CRISPR/Cas9

The single guide RNA (sgRNA) for *Ednrb* was designed to target the sequence 5’-AGCCGGTGCGGACGCGCCTTGG-3’ on exon 2 of *Ednrb*. sgRNA for *Rosa26* was designed to target the sequence 5’- GACTCCAGTTGCAGATCACG -3’ on exon 1 of *Rosa26*. sgRNAs were transcribed *in vitro* using a MEGAshortscript T7 Transcription Kit (Thermo Fisher Scientific, Carlsbad, CA, USA) from synthetic double-stranded DNAs obtained from Integrated DNA Technologies, IA, USA or Thermo Fisher Scientific. The pCas9-poly was formally constructed and deposited into the Addgene repository (ID #72602; www.addgene.org/CRISPR). mRNA was transcribed *in vitro* using a mMESSAGE mMACHINE T7 Ultra Kit (Thermo Fisher Scientific) from linearized plasmids and was purified using a MEGAClear kit (Thermo Fisher Scientific).

Super ovulation was induced in female GK and F344 rats by injecting with 150 U/kg pregnant mare serum gonadotropin and 48h later 75 U/kg human chorionic gonadotropin. The super-ovulating female rats were mated with male rats of the same strain. The female rats confirmed to have copulatory plugs were euthanized by the inhalation of CO_2_ the following morning and pronuclear-stage embryos were collected. The sgRNA for *Ednrb* and *Rosa26* and Cas9 mRNA were introduced into embryos using a NEPA21 Super Electroporator (Nepa Gene, Ichikawa, Japan). The embryos that developed into 2-cell embryos were collected and transferred into the oviducts of female surrogate SD rats anesthetized with isoflurane.

### CRISPR-mediated mutation analysis

DNA samples from the puerperal offspring of the GK and F344 strains were prepared from tail tips. Primer set 2 (F-GGCGCGCAAACTTAACTTAC and R-GGGACCATTTCTCATGCACT) flanking a 583-bp sequence, including the targeting sequence of sgRNA on *Ednrb*, and primer set 3 (F- TGCTCTCCAAAAGTCGGTTT and R-CCCAGGTGAGTGCCTAGTCT) flanking a 391-bp sequence, including the targeting sequence of sgRNA on *Rosa26*, were designed to amplify the targeting sequence. PCR was performed in a total volume of 15 μl under the following conditions: 1 cycle at 94°C for 3 min; 35 cycles of 94°C for 30 s, 60°C for 1 min, and 72°C for 45 s; and 1 cycle at 72°C for 3 min. The PCR products were then directly sequenced using a BigDye Terminator v3.1 Cycle Sequencing Kit and the standard protocol for an Applied Biosystems 3130 DNA Sequencer (Thermo Fisher Scientific).

### Acetylcholine esterase (AChE) staining

The whole intestine (from pylorus to anus) was dissected from the 10-day-old pups of F_2_(F344 × GK) and genome-edited F344 rats. After the attachments and contents were removed, the intestine was subjected to AChE whole-mount staining [26]. Visualization of the enteric ganglia and measurement of the aganglionic length were conducted under a microscope. The ratio of the affected length to the length of the large intestine was calculated to determine the severity of aganglionosis as described in a previous paper [23].

## Results

### Selection of a rat strain possessing a similar haplotype in the responsible region of Chr 2 on order to create a new rat model that shows the severe symptoms of aganglionosis

Previous studies identified a QTL on Chr 2 as responsible for controlling the severity of aganglionosis in the rat model [24,25]. It has been indicated that several SNPs on three genes, *Gdnf*, *Slc45a2*, and *Ptger4*, located in this region constitute different haplotypes between the rat strains showing mild vertsus severe symptoms [25]. To verify whether the haplotype of these SNPs modifies the symptoms caused by *Ednrb* mutations and to create a new rat model that displays the severe symptoms, we investigated the sequence polymorphisms of the recorded rat strains in the RGD to identify any rat strains possessing a haplotype similar to that of the AGH rat. According to the information in the database, the GK rat strain was found to display the most similar haplotype to that of the AGH rat among registered inbred strains. After obtaining the GK/Slc rat, we determined its haplotype (Table 1) using the method described in a previous paper [25]. The positions of the genes and microsatellite markers are based on information from *Rattus norvegicus* (assembly Rnor_5.0).

**Table 1:** The SNPs in the GK, AGH, F344, and LEH inbred strains.

Gene	SNP Location	F344/LEH	AGH/GK
*Ptger4*	g.73985633, exon 1	T/T	C/T
	g.73986958, promoter	G/G	A/G
	g.73987571, promoter	C/C	T/C
*Gdnf*	g.76896910, promoter	C/C	T/T
	g.76897291, promoter	C/C	T/T
	g.76901040-76901042, intron 1	TTA/TTA	-/TAA
	g.76901607, intron 1	G/G	A/A
	g.76901863, intron 1	-/G	-/-
	g.76917833-76917835, intron 2	AAG/AAG	-/-
	g.76918613, intron 2	C/C	A/A
	g.76918959, intron 2	G/A	G/G
	g.76919179, intron 2	C/C	T/T
*Slc45a2*	g.83715441, exon 2	G/G	A/G
	g.83717275, exon 1	C/C	T/C
	g.83717367, exon 1	A/A	T/T
	g.83717975, promoter	A/A	-/-
	g.83718063, promoter	G/G	A/A
	g.83718133, promoter	G/G	A/A

### F_2_(F344 × GK)-*Ednrb*^*sl*^ progeny with the GK haplotype in the responsible region on Chr 2 experience embryonic death

**Fig 1:**
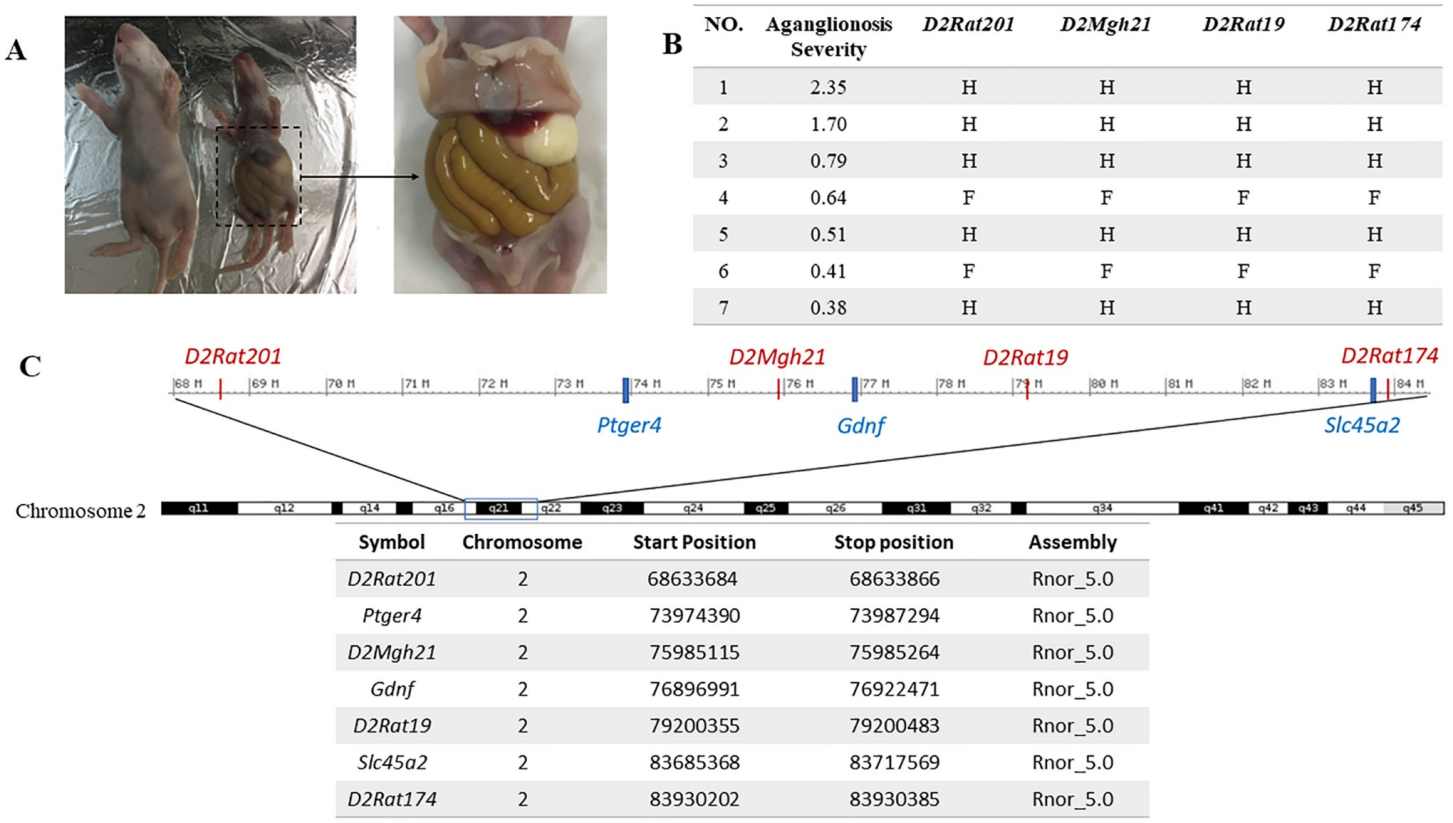
The haplotype and phenotype of F_2_(F344 × GK)-*Ednrb*^*sl/sl*^ progeny. **(A)** The 10-day-old offspring of F_2_(F344 × GK). Right, a pup carrying the *Ednrb* mutation showes very severe symptom. This pup has an obviously inflated intestine and a hypoplasia. Left, a healthy littermate. **(B)** The aganglionosis ratio and haplotype for 7 mutant F_2_ progeny. The aganglionosis severity was calculated as the aganglionosis ratio (agangliononic length divided by large intestine length). Four microsatellites located in the responsible region were selected for the genotyping of the F_2_(F344 × GK) generation, and the genotypes of microsatellite loci are listed in the table. F indicates the homozygous genotype of the F344 strain. H indicates the heterozygous genotype of GK and F344 rats. No mutant progeny that processed the homozygous genotype of the GK strain were found. **(C)** A schematic of the identified region on Chr 2. The candidate genes are marked in blue and the microsatellites are marked in red. The location of each marker and candidate gene is listed.

To verify that the haplotype of responsible region in the GK rat also causes the severe symptoms that are seen with the mutant *Ednrb*, we produced F_2_(F344 × GK) progeny by mating GK females to F344-*Ednrb*^*sl/+*^ males and crossing the heterozygous F_1_ progeny. Fifty 10-day-old F_2_(F344 × GK) progeny were harvested. After genotyping and histological analysis, we found that F_2_(F344 × GK)-*Ednrb*^*sl/sl*^ progeny presented various extent of aganglionosis, including the severe symptoms similar to those seen in the AGH-*Ednrb*^*sl/sl*^ rat (Fig 1A and 1B), suggesting that the genetic background of the GK inbred strain exacerbates the extent of aganglionosis severe. To easily determine the haplotype of the responsible region in the F_2_ progeny, we selected 4 microsatellite markers (*D2Rat201*, *D2Rat174*, *D2Rat19*, and *D2Mgh21*) located in the responsible region including the three candidate genes (Fig 1C). After performing genotyping, we found that two progeny, progeny 1 and progeny 2, which showed the severe symptoms of aganglionosis possessed heterozygous haplotype in the responsible region, suggesting that the GK rat may carry the modifier gene(s) responsible for the severity of aganglionosis in the responsible region. Other F_2_ progeny showing much milder symptoms of aganglionosis possessed either the heterozygous or the F344-homozygous haplotype (Fig 1B). Notably, no GK-homozygous F_2_ progeny were obtained (Fig 1B). Therefore, we hypothesized that the progeny carrying the GK-homozygous haplotype in the responsible region displayed more severe symptoms that caused them to die in the prenatal stages if they carried the *Ednrb* mutation. To test this hypothesis, we compared the number of F_2_ progeny for each haplotype of the responsible region in all 50 F_2_ progeny, including the wildtype rats, the heterozygous *Ednrb*^*sl*^ rats, and the homozygous *Ednrb*^*sl*^ rats (Table 2). In 43 healthy F_2_ progeny possessing either the wildtype or the heterozygous *Ednrb*^*sl*^ mutation, the numbers of F344-homozygous, heterozygous, and GK-homozygous genotypes of each microsatellite followed an approximately 1:2:1 ratio, in agreement with the Mendelian rule. However, F_2_ progeny possessing the GK-homozygous genotype were not obtained in the homozygous *Ednrb*^*sl*^ mutation. We further investigated 29 embryos of the F_2_(F344 × GK) generation at the E16 stage. The results were consistent with those for 10-day-old offspring, i.e., no F_2_(F344 × GK)-*Ednrb*^*sl/sl*^ embryos carried the GK haplotype (data not shown). These data strongly suggest that the combination of the homozygous *Ednrb*^*sl*^ mutation and the GK haplotype in the responsible region on Chr 2 are what cause embryonic death.

**Table 2:** The number and ratio of 10-day-old pups possessing the genotype of each microsatellite in the F_2_(F344 × GK) generation.

Genotype of Microsatellite Genotype of *Ednrb* gene	*D2Rat201*			*D2Mgh21*			*D2Rat19*			*D2Rat174*		
	F/F(%)	F/G(%)	G/G(%)	F/F(%)	F/G(%)	G/G(%)	F/F(%)	F/G(%)	G/G(%)	F/F(%)	F/G(%)	G/G(%)
*Ednrb*^*+/+*^	6(31)	8(42)	5(26)	6(31)	8(42)	5(26)	6(31)	8(42)	5(26)	7(37)	7(37)	5(26)
*Ednrb*^*sl/+*^	7(29)	12(50)	5(21)	6(25)	13(54)	5(21)	6(25)	14(58)	4(17)	6(25)	14(58)	4(17)
*Ednrb*^*sl/sl*^	2(28)	5(71)	0(0)	2(28)	5(71)	0(0)	2(28)	5(71)	0(0)	3(43)	4(57)	0(0)

### Targeted disruption of *Ednrb* by genome editing in GK rats

The absence of F_2_(F344 × GK)-*Ednrb*^*sl/sl*^ progeny carrying the GK haplotype in the responsible region led us to examine whether the disruption of the *Ednrb* can cause embryonic death in the GK inbred strain and can cause the related embryonic phenotype. To verify this hypothesis, we employed the CRISPR/Cas9 system to knockout the *Ednrb* in the GK strain. The genome editing experiments were also performed in the F344 rat as a control. The sgRNA was designed targeting on exon 2 of *Ednrb* in the rat. The technique for animal knockout system by electroporation (TAKE) method was used to produce the genome-edited rat [27]. After introducing the Cas9 mRNA and sgRNA-*Ednrb* into the zygotes collected from the F344 strain by electroporation, twenty-three 2-cell embryos were transferred into the oviducts of one surrogate rat, and 4 pups were obtained (Table 3). After the targeted sequence of these four offspring were analyzed, two of the offspring were identified as bi-allelic knockout, one carried a 14-bp deletion and a 16-bp deletion, and another carried a 2-bp deletion and a 13-bp deletion in the two alleles. These deletions all caused the frame shift. The other two littermates were found to carry the wildtype allele. The intestines of these offspring were collected at postnatal day 10 and subjected to whole-mount AChE staining. The two offspring carrying the deleted alleles were confirmed to show aganglionosis in a small proportion of the large intestine (0.14 and 0.18). The results from the F344 rats verified the efficiency of the TAKE method and the sgRNA. Then, zygotes collected from the GK rat were transfected with Cas9 mRNA and sgRNA-*Ednrb* using the same method and eighty-eight 2-cell embryos were transferred into the oviducts of 4 surrogate female rats. However, only one female rat gave birth, to 2 pups which died soon after birth, and were eaten by the surrogate mother. Only a part of one tail was left. We examined the targeted sequence of genome DNA extracted from the tail clip, and two kinds of alleles were identified. One allele was the wildtype, and the other carried a 35-bp deletion. After dissecting the uterus of the surrogate rats, we found tiny implantation sites, indicating that most of the embryos had died after implantation. These results suggest that the disruption of *Ednrb* caused embryonic death. However, we performed genome editing on an unrelated gene, *Rosa26*, in the GK rat using the same procedure and conditions to test the above hypothesis. It was found that only 2.8% of the transferred embryos survived to birth, and both of these were heterozygote/mosaic (Table 3). The low reproduction rate of the *Rosa26*-modified GK rats suggests that the genome editing procedure itself causes embryonic death in the GK rat, indicating that it is impossible to use the genome editing strategy to verify that *Ednrb* knockout causes the embryonic death in the GK rat.

**Table 3:** The genome-edited offspring in the F344 and GK strain.

Strain	Gene	Number of transferred embryos	Number of litters (%)^a^	Number of offspring (%)^b^	WT offspring	Bi-allelic KO (Phenotype)	Heterozygote/Mosaic (Phenotype)
GK	*Ednrb*	88	1 (25%)	2 (2.3%)	0	0	100% (Early infant death)
	*Rosa26*	72	2 (66.7%)	2 (2.8%)	0	0	100%
F344	*Ednrb*	23	1 (100%)	4 (17.4%)	0	50% (Mild aganglionosis)	50% (Normal)

^a^ Calculate from the total number of female rats used as surrogate.

^b^ Calculate from the total number of transferred embryos.

## Discussion

*Ednrb*, a critical gene in the development of HSCR, encodes a G-protein-coupled receptor for the ligand of EDN3 and is expressed primarily by migrating enteric neural crest-derived cells (ENCCs) [1]. Generally, mutations of this gene cause pigmentary abnormalities, deafness and aganglionosis in humans and many other higher organisms [5,28]. These common phenotypes were also observed in our previous rat models, which carry a spontaneous null mutation in *Ednrb* [23]. This null mutation caused severe aganglionosis in one of these rat strains, the AGH strain, leading to a lethality during the weaning stage, which is distinct from the other rat models. In this study, we also found that the *Ednrb* mutation caused much greater lethality in the embryonic stage in the GK inbred rat strain. A similar phenotype was found in studies of the *Ednrb*^*s-1Acrg*^ mutation, a 1.3-cM deletion on Chr 14, flanking the *Ednrb* gene in the mouse genome [29]. This deletion also causes embryonic death in the early gestational period, and the homozygous embryos displayed gross morphological defects including poorly developed head folds and caudal truncation. The number of homozygous embryos was distinctly decreased at E11.5, and there were no homozygous embryos after E12.5 during embryogenesis. Similarly, another *Ednrb*^*s-36Pub*^ mutation, which partly overlaps the *Ednrb*^*s-1Acrg*^ deletion, leads the death of homozygous offspring at birth, which are consistent with the phenotype of the *Ednrb*^*s-1Acrg*^ heterozygous mice [30]. Studies of the genes and loci on this large deletion have proved the embryonic lethality in this case to require the loss of multiple genes and to involve complicated genetic mechanisms that remain unclear [30].

In our study, we also found that the embryonic death was caused by the mutation of the *Ednrb* gene in the GK inbred strain. As the GK rat was confirmed to carry a haplotype in the responsible region on Chr 2 similar to that of the AGH rat presenting severe aganglionosis, we expected that the *Ednrb*-null mutation to lead to the similar severe symptoms in the GK inbred strain. But even though some of the F_2_(F344 × GK)-*Ednrb*^*sl/sl*^ progeny displayed severe aganglionosis, no mutant progeny with the GK-homozygous haplotype in the responsible region were reproduced. To verify the embryonic death of the *Ednrb*-mutant GK rats, we conducted genome editing to generate *Ednrb*-null GK rats. Editing of the *Ednrb* gene caused the production of fewer offspring than for the control rat, F344. However, the genome editing of an unrelated gene, *Rosa26*, in GK rats with the same methods caused the production of similarly few offspring, suggesting that the genome editing itself caused the embryonic death in the GK rat. It is unknown at present what procedure in the genome editing causes the embryonic death in the GK rat.

The GK strain is a classical rat model of non-insulin-dependent diabetes mellitus (NIDDM) [31]. These animals always present symptom of type 2 diabetes, such as fasting hyperglycemia, impaired insulin secretion in response to glucose, and insulin resistance; however, these symptoms all appear at the young adult stage of 8 weeks old in the GK/Slc inbred strain [32], suggesting that the diabetes is not related to the embryonic death. Several studies have already identified a series of genetic loci that relate to the diabetes phenotype of the GK inbred strain [31,33,34]. These loci are located on multiple chromosomes, but none of them overlaps the responsible region for the modifier of the aganglionosis phenotype on Chr 2. These data add evidence that the embryonic lethal phenotype is independent of the diabetes symptoms in the GK inbred strain.

The responsible region on Chr 2 includes another critical gene, *Gdnf*, which relates to the survival, proliferation, migration, and differentiation of ENCCs [25,35]. Some studies have found that patients with HSCR carry heterozygous mutation on the *Gdnf* and that *Gdnf*^*+/−*^ mice also show an approximately 50% reduction in ENCC number [12,36]. Using *in vitro* studies, we also elucidated that the combination of the AGH-type SNPs in the *Gdnf* promoter reduces *Gdnf* expression [37]. Other studies have revealed that *Gdnf*^*-/-*^ animals always die within 24 h after birth and that after the first 24 h, the *Gdnf*^*−/+*^ animals died, too [38–40]. Thus, considering the role of *Gdnf* in the development of ENS, we speculate that *Gdnf* plays a critical role in modifying the symptoms caused by the *Ednrb* mutation in our rat models. However, the AGH-*Ednrb*^*sl*^ rats, whose haplotype in the responsible region on Chr 2 is mostly consistent with that of the GK strain, were able to survive until 3 weeks old, whereas GK rats with a mutation of *Ednrb* showed embryonic death. We think that some other modifiers relating to the early embryonic death may exist in the genetic background of the GK rat. We suggest that the GK rat can be valuable as a laboratory animal model for studying the interaction of genes involved in HSCR disease and embryogenesis. Further studies that uncover the modifier genes and genetic mechanisms in the GK rat strain promise to facilitate the understanding of the regulatory network of genes for the embryogenesis and development of ENS and HSCR.
